# Point-of-Care Lung Ultrasound for COVID-19: Findings and Prognostic Implications From 105 Consecutive Patients

**DOI:** 10.1177/0885066620988831

**Published:** 2021-02-04

**Authors:** Kosuke Yasukawa, Taro Minami, David R. Boulware, Ayako Shimada, Ernest A. Fischer

**Affiliations:** 1 Division of Hospital Medicine, Department of Medicine, 8405 MedStar Washington Hospital Center, Washington, DC, USA; 2 Division of Pulmonary, Critical Care, and Sleep Medicine, Care New England Medical Group, Pawtucket, RI, USA; 3 Division of Pulmonary, Critical Care, and Sleep Medicine, Department of Medicine, the Warren Alpert Medical School of Brown University, Providence, RI, USA; 4 Division of Infectious Diseases and International Medicine, Department of Medicine, 5635 University of Minnesota, Minneapolis, MN, USA; 5 Division of Biostatistics, Department of Pharmacology and Experimental Therapeutics, Sidney Kimmel Medical College, Thomas Jefferson University, Philadelphia, PA, USA; 6 Division of Hospital Medicine, Department of Medicine, MedStar Georgetown University Hospital, Washington, DC, USA

**Keywords:** point-of-care ultrasound, lung ultrasound, COVID-19, coronavirus, viral pneumonia

## Abstract

**Background:**

The prognostic value of point-of-care lung ultrasound has not been evaluated in a large cohort of patients with COVID-19 admitted to general medicine ward in the United States. The aim of this study was to describe lung ultrasound findings and their prognostic value in patients with COVID-19 admitted to internal medicine ward.

**Method:**

This prospective observational study consecutively enrolled 105 hospitalized participants with COVID-19 at 2 tertiary care centers. Ultrasound was performed in 12 lung zones within 24 hours of admission. Findings were assessed relative to 4 outcomes: intensive care unit (ICU) need, need for intensive respiratory support, length of stay, and death.

**Results:**

We detected abnormalities in 92% (97/105) of participants. The common findings were confluent B-lines (92%), non-homogenous pleural lines (78%), and consolidations (54%). Large confluent B-lines, consolidations, bilateral involvement, and any abnormality in ≥ 6 areas were associated with a longer hospitalization and need for intensive respiratory support. Large confluent B-lines and bilateral involvement were also associated with ICU stay. A total lung ultrasound score <5 had a negative predictive value of 100% for the need of intensive respiratory support. A higher total lung ultrasound score was associated with ICU need (median total 18 in the ICU group vs. 11 non-ICU, p = 0.004), a hospitalization ≥ 9d (15 vs 10, p = 0.016) and need for intensive respiratory support (18 vs. 8.5, P < 0.001).

**Conclusions:**

Most patients hospitalized with COVID-19 had lung ultrasound abnormalities on admission and a higher lung ultrasound score was associated with worse clinical outcomes except death. A low total lung ultrasound score (<5) had a negative predictive value of 100% for the need of intensive respiratory support. Point-of-care ultrasound can aid in the risk stratification for patients with COVID-19 admitted to general wards.

## Introduction

The number of patients infected with severe acute respiratory syndrome-coronavirus-2 (SARS-CoV-2) continues to increase in the United States and globally. Initial reports from China reported that approximately 14% with coronavirus disease 2019 (COVID-19) developed severe illness requiring hospitalization, and 5% developed critical illness.^1^ Among hospitalized patients with COVID-19, the primary manifestation is viral pneumonia,^2^ and among those with pneumonia, 20-42% have developed acute respiratory distress syndrome (ARDS).^3^

Multiple diagnostic tools exist for diagnosing pneumonia. Computed tomography (CT) of the chest is a sensitive imaging modality to diagnose COVID-19 and monitor disease progression.^4–6^ COVID-19 pneumonia imaging typically shows bilateral multilobar ground-glass opacities with a peripheral or posterior distribution.^7^ Lesions may progress to consolidative opacities as the pneumonia progresses.^7^ Despite the utility of CT, its use as a diagnostic modality is limited by multiple factors including the need for transport of infectious or unstable patients and need for disinfection of radiology facilities afterward. In addition, CT may not be readily available in rural health centers or other resource-limited settings. An alternative radiologic technique is point-of-care ultrasound (POCUS) of the lung for the diagnosis and monitoring of patients with COVID-19.^8,9^ Lung ultrasound is a suitable imaging modality in COVID-19 pneumonia as the lung lesions are predominantly peripheral. Previous studies have shown lung ultrasound can aid COVID-19 diagnosis.^10–13^

Most studies on ultrasound in patients with COVID-19 involved small cohorts. Larger studies on the use of ultrasound in the emergency department,^14,15^ pulmonary service,^16^ and medical ward, or intensive care^17^ indicated that lung ultrasound findings early in the diagnosis were associated with worse clinical outcomes. No large study has been performed in patients with COVID-19 admitted to general medicine ward in the United States, and none has attempted to demonstrate predictive ability of ultrasound findings. The present study was performed to determine the prognostic value of findings at the time of hospital admission in a prospective cohort of consecutive COVID-19 patients.

## Methods

### Study Design, Setting, and Population

We conducted a prospective observational cohort study at 2 tertiary care hospitals, MedStar Georgetown University Hospital (MGUH) and MedStar Washington Hospital Center (MWHC), in Washington DC. Over a 3-week period during the peak of the first wave of infections in our region (April 18 to May 9, 2020 at MWHC and from 21 to May 10, 2020 at MGUH), we screened adults aged ≥ 18 years for study eligibility who were admitted to a hospital medicine service. Patients were included if they had a positive polymerase chain reaction (PCR) for SARS-CoV-2 and were approached within 24 hours of admission. During the study period, patients generally had PCR results within 4 hours of collection. Patients were excluded if they were unable to provide consent, were unable to consent in English, pregnant, or had known structural lung abnormalities. We collected demographics, comorbidities, presenting symptoms, laboratory data, radiologic information, and outcomes during the hospitalization. Written informed consent was obtained from each participant. This study was approved by the MedStar Research Institutional Review Board(IRB ID: STUDY00002254).

### Point-of-Care Lung Ultrasound Examination

**Figure 1.**
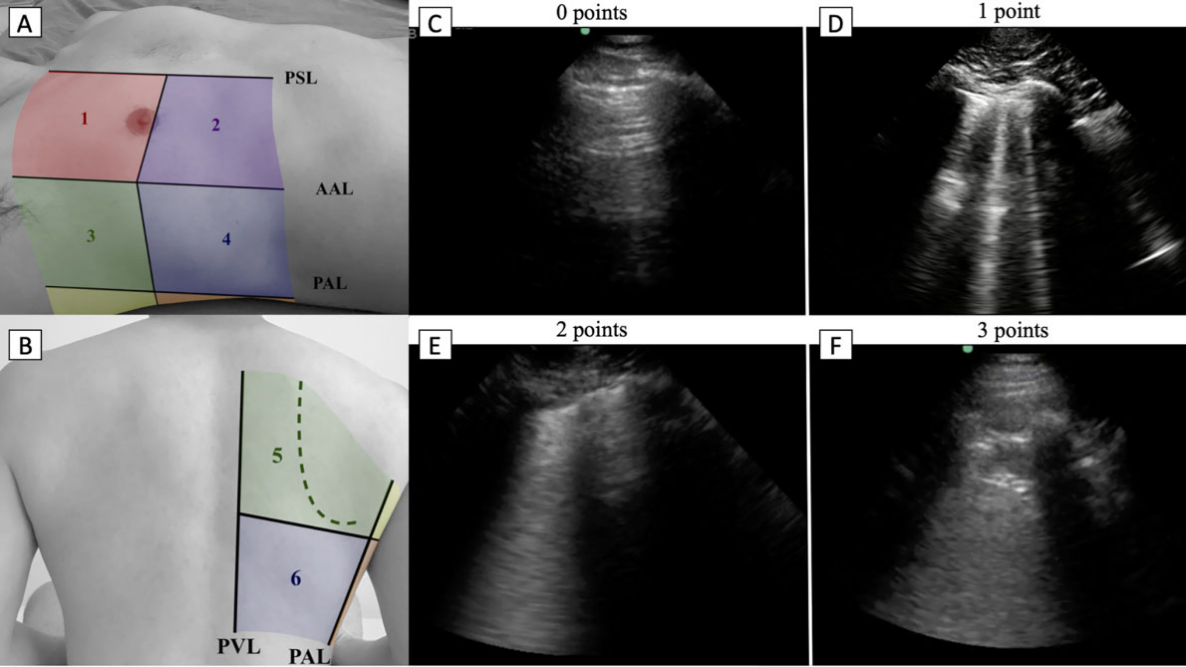
Lung ultrasound zones and scoring. A and B, Zones of lung ultrasound. PSL parasternal line, AAL anterior axillary line, PAL posterior axillary line, PVL paravertebral line. Examples of lung ultrasound findings according to lung ultrasound point. C, Normal aeration of the lung. D, Three well differentiated B-lines, E, Confluent B-line, F, > 1 cm consolidation.

An M-Turbo ultrasound machine and a 1- to 5-MHz phased array probe (FUJIFILM SonoSite, Bothell, WA) were used for the majority of the examinations (86 patients). SonoSite Edge II with a 1- to 5- MHz phased array probe (FUJIFILM SonoSite, Bothell, WA) and Philips Lumify with a 1- to 4-MHz phased array probe (Philips, Reedsville, PA) were used for 15 patients and 4 patients, respectively. Lung ultrasound was performed in 12 zones (**Figure 1**).^18–20^ Each intercostal space of upper and lower parts of the anterior, lateral, and posterior regions of the right and left chest wall was examined. Within each region, the worst ultrasound finding detected was considered as characterizing the region. The duration of each ultrasound examination was approximately 10-15 minutes. Ultrasound probe and machine were cleaned with recommended disinfectant before and after each use.

Ultrasounds were performed by authors EF and KY at MGUH and MWHC, respectively. Both scanners have the Society of Hospital Medicine/American College of Chest Physicians POCUS Certificate of Completion and teach POCUS locally and nationally. Additionally, KY successfully completed the Examination of Special Competence in Critical Care Echocardiography of the National Board of Echocardiography (NBE). TM instructs nationally and internationally and director of several courses. TM also has completed the Examination of Special Competence in Adult Echocardiography by the NBE.

### Lung Ultrasound Score and Findings

Each lung zone was evaluated for the following findings, and listed by presumed increasing severity:

B line spectrum abnormalities

- pathologic B lines (≥ 3 well-defined B lines)
- confluent B line(s), small (<1 cm) and large (>1 cm)
- white lung (any increased echogenicity below the pleural line, covering the entire rib space and persisting during the entire respiratory cycle)

Pleural line spectrum abnormalities

- non-homogenous pleural line
- consolidation, small (< 1 cm) “subpleural” or large (> 1 cm in any dimension)

For the lung ultrasound score, 0-3 points were allocated to each zone (Figure 1) according to the following:

0 - the presence of lung sliding with A-lines or fewer than 2 well-defined B lines
1 - ≥ 3 well-defined B-lines
2 - confluent B-line(s), large or small, white lung with, or small (< 1 cm) consolidation
3 - consolidation >1 cm.

A total lung ultrasound score was calculated as the sum of scores for each of the 12 zones, with a possible range of 0 to 36.

### Patient Outcomes

The following clinical outcomes were recorded for each enrolled patient: intensive care unit (ICU) stay, the overall length of stay, and death from any cause. An additional composite outcome of “intensive respiratory support” included those requiring high-flow nasal cannula, non-rebreather, or mechanical ventilation. We planned to compare the frequencies of lung ultrasound scores by zone, the total lung ultrasound score, and the individual findings (i.e., small confluence, large consolidation) to each of these clinical outcomes to determine if any were over-represented. Additionally, we wanted to see if the lung ultrasound scores or individual findings had any power in predicting outcomes.

### Lung Scoring

Each lung ultrasound clip was scored independently by EF and KY. Then all 1260 images were reviewed again jointly by EF, TM, and KY, regardless of agreement, to arrive at a final consensus description and score for each clip.

### Statistical Analyses

Lung zone ultrasound findings and scores were compared by outcome with the chi-square test, Fisher’s exact test, and Kruskal-Wallis test, as appropriate with SAS software (SAS Institute Inc., Cary, NC). Other analyses were conducted in R.^21^

Agreement between the raters was evaluated with Cohen’s Kappa coefficient via the “vcd” package before calculating final scores.^22^ The LOS was compared to a lung ultrasound score made dichotomous (using 66% cutpoint) and using survival analysis with logrank testing.^23^

Logistic regression models were built to predict patients’ outcomes based on ultrasound findings or scores. Model operating characteristics were calculated using the ROCR package.^24^ A priori 70% of the data was selected to train models, with the remaining 30% used for testing. Models were refined, excluding zones or findings that did not contribute to the outcome prediction (based on the regression coefficient having a p-value <0.1) to improve the prediction. The same 70% and 30% subsets were used for all derivations. Sensitivity and specificity are based on the predictions of the model on the test set alone. Positive and negative predictive values were based on the model performance on all patients. No data were censored. Tests were performed at a significance level of 0.05 except for the logistic regression model derivation for which we used a more stringent level of 0.01 for significance.

## Results

### Participant Characteristics

During the study period, 229 patients with COVID-19 (171 at MWHC and 58 at MGUH) were admitted to the hospital medicine services at MWHC and MGUH. We enrolled 113 participants. Informed consent could not be provided by 59 non-English speakers, 49 were unable to consent 3 declined to consent, 1 was a prisoner and 4 had exclusion criteria (1 pregnancy, 3 structural lung disease). Of the 113 enrolled, 105 participants underwent a LU within 24 hours of admission. LU could not be completed within 24 hours of admission in 8 patients due to: transfer of care (1), significant dyspnea (1), or sonographer’s clinical duties (6). The enrollment is summarized in Supplemental Figure 1.

The demographic and clinical characteristics of the enrolled participants are provided in **Table 1**. The median age was 57 years (Interquartile range [IQR], 48 to 68), 65 patients (62%) were male, 65% were black, and 34% were Hispanic. Overall, 62 (59%) had hypertension, and 30 (29%) had diabetes mellitus. Obesity was common (52%), and the median BMI was 29.8 (IQR, 26.7 to 34) kg/m.^2^ The median days since the symptom onset was 6 (IQR, 4 to 8). The most common presenting symptoms were dyspnea (76%), cough (76%), and fever (62%). During the study period, remdesivir or steroid therapy were not utilized; however, 13 (12%) participants received convalescent plasma therapy, and 5 (5%) received tocilizumab.

**Table 1.** Demographic and Clinical Features of Patients With COVID-19 at Time of Hospital Admission Associated With Need for Intensive Respiratory Support During the Hospitalization.

Characteristic	All patients (N = 105)	No intensive respiratory support (N = 72)	Requiring intensive respiratory support (N = 33)
**Age**, years	57 (48, 68)	56 (48, 64)	62 (48, 72)
Men	65 (62)	43 (60)	22 (67)
**Race**			
Black	65 (62)	45 (63)	20 (61)
Hispanic	34 (32)	22 (31)	12 (36)
White	5 (5)	4 (6)	1 (3)
Asian	1 (1)	1 (1)	0 (0)
**Comorbidities**			
Hypertension	62 (59)	48 (67)	14 (42)
Obesity (BMI ≥ 30)	52 (50)	34 (47)	18 (55)
Diabetes mellitus	30 (29)	19 (26)	11 (33)
End-stage renal disease	12 (11)	7 (10)	5 (15)
Congestive heart failure	7 (7)	4 (6)	3 (9)
Cancer	7 (7)	5 (7)	2 (6)
Asthma	6 (6)	5 (7)	1 (3)
Chronic obstructive lung disease	2 (2)	1 (1)	1 (3)
**Symptoms**			
Days since symptom onset	6 (4 - 8)	7 (4-8)	5 (4–8)
Dyspnea	76 (72)	50 (69)	26 (79)
Cough	76 (72)	51 (71)	25 (76)
Fever	62 (59)	40 (56)	22 (67)
Asymptomatic	7 (7)	7 (10)	0 (0)
Body Mass index kg/m^2^	29.8 (26.7, 34)	29.4 (26.4, 34.2)	30.7 (27, 33.6)
Oxygen saturation on room air	93 (90, 97)	95 (92, 97)	91 (88, 93)
**Initial laboratory measures**			
White blood cells, x 10^9^ /L	6.4 (4.5, 8.1)	5.9 (4.5, 7.6)	7.2 (4.7, 8.4)
Lymphocyte count, k/uL	1200 (800, 1600)	1250 (900, 1600)	900 (600, 1300)
D-dimer, mcg/mL	0.98 (0.52, 1.92)	0.8 (0.45, 1.65)	1.4 (0.87, 2.9)
Ferritin, ng/mL	561 (247, 1185)	361 (238, 1082)	940 (561, 1756)
C-reactive protein, mg/L	74 (22, 152)	57 (14, 107)	156 (67, 183)
Lactate dehydrogenase, u/L	360 (266, 469)	298 (256, 414)	418 (342, 541)
Creatine kinase U/L	164 (83, 334)	136 (73, 306)	194 (137, 680)
Troponin, abnormal	24 (23)	13 (18)	11 (33)
ESR >85 mm/hr	29 (28)	17 (24)	12 (36)
**Treatment**			
Convalescent plasma therapy	13 (12)	1 (1)	12 (36)
Tocilizumab	5 (5)	0	5 (15)
**Outcome**			
Intensive care	25	0 (0)	25 (76)
Mechanical ventilation	13 (12)	0 (0)	13 (39)
Death	9 (9)	0 (0)	9 (27)

Values are N (%) or median (IQR). Intensive respiratory support defined as requiring high flow oxygen via nasal cannula, non-rebreather mask, or mechanical ventilation during admission. Of note all deaths occurred in the intensive respiratory support group.

Of the 105 participants, 7 (6.6%) were asymptomatic (admitted for other problems, remained on room air, and did not have fever, cough, dyspnea, or other COVID-19 related symptoms), 65 (62%) were symptomatic but did not require IRS and 33 (31%) required IRS, 25 (24%) required intensive care, 13 (12%) required mechanical ventilation, and 9 (8.6%) died during the hospitalization.

#### Lung ultrasound findings

A total of 1260 lung ultrasound images were collected from the 105 participants, 12 lung zones each. Abnormal findings were detected in 97 (92%) participants, with 80 (76%) having abnormal findings in bilateral lungs. Confluent B-lines were the most common finding (92%) along with a non-homogenous pleural line (78%). Lung consolidation was observed in 57 patients (54%) with large consolidation in 33 (31%). Large consolidations were found mostly in the posterior bases (33 large consolidations in L6 or R6 out of 53 total large consolidations). Discrete B-lines were seen in 17 patients (16%), and discreet B-lines without any confluence was rare (<1% of all images). The median number of lung zones with abnormalities per patient was 6 (IQR, 3 to 9).

The median total lung ultrasound score was 12 (IQR, 6 to 18, max 25). Inter-rater reliability for the score was 0.95 (95% CI 0.94-0.97) with only 34 (2.7%) total disagreements and only 0.55% (7/1260) scores changed before arriving at the final consensus. The score distribution by zone is pictorially displayed in Supplementary Figure 2.

#### Lung findings by clinical outcome

**Figure 2.**
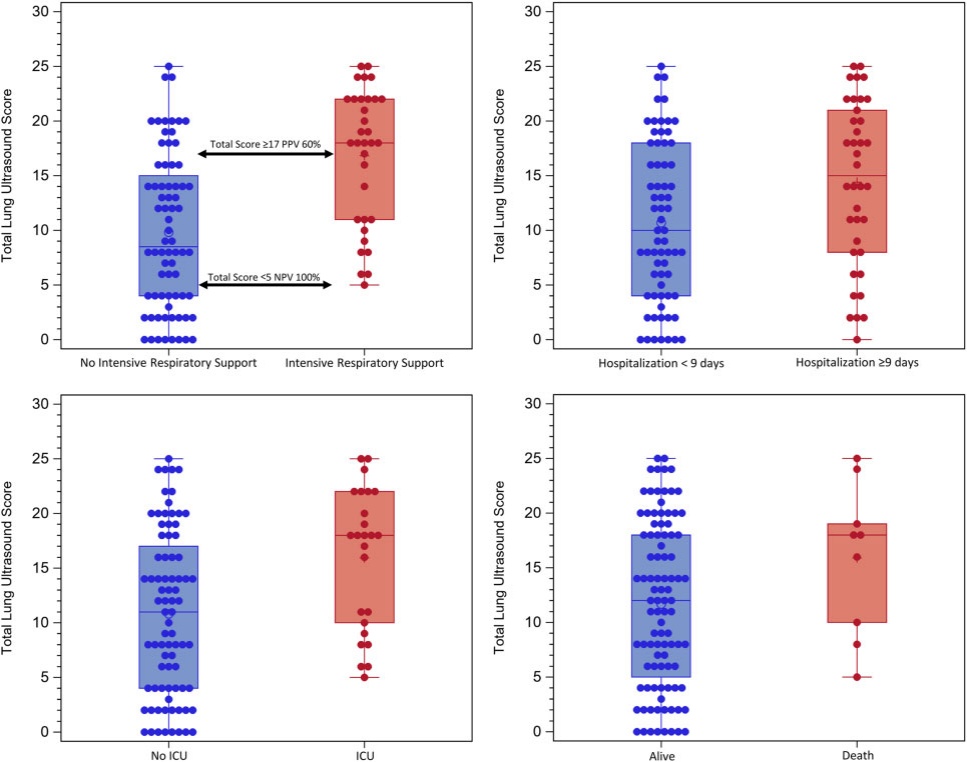
Boxplots of the total lung ultrasound score: (1) Asymptomatic or non-intensive respiratory support vs. intensive respiratory support (top left), (2) Length of stay less than 9 days vs. greater than/equal 9 days (top right), (3) No ICU vs. ICU (down left), and (4) Alive vs. death (down right).

Multiple abnormalities were over-represented in the predefined outcome groups except for the outcome of death, which had no associations. A higher total lung ultrasound score was associated with need for the ICU (median total lung ultrasound 18 in the ICU group vs. 11 non-ICU group; p = 0.004), a length of stay ≥ 9d (15 vs 10, p = 0.016) and a need for intensive respiratory support (18 vs. 8.5, P < 0.001, see **Figure 2**). A total lung ultrasound score <5 had a negative predictive value of 100% for the need of intensive respiratory support. Bilateral findings and any large (>1 cm) confluent B-lines were also seen more frequently in these outcomes. Small consolidations were over-represented in the length of stay ≥ 9d (p = 0.006) and intensive respiratory support groups (P = 0.004), but not in death or ICU groups. Surprisingly, large consolidations were only over-represented in the intensive respiratory support group (p = 0.036), but not hospitalization duration ≥ 9d, death, or ICU groups. The summary of findings by outcome group is summarized in **Table 2**.

**Table 2.** Lung Ultrasound Findings and Outcome in Hospitalized Patients Admitted With COVID-19.

	All patients n = 105	Asymptomatic/non-intensive respiratory support n = 72	Intensive respiratory support n = 33	p - value	Length of stay < 9 days, n = 52	Length of stay 9 days, n = 53	p - value
Abnormal lung ultrasound	97 (92)	64 (89)	33 (100)	0.054	60 (90)	37 (97)	0.253
Distribution							
Bilateral	80 (76)	47 (65)	33 (100)	**<0.001**	47 (70)	33 (87)	0.054
Affected areas ≥ 6	56 (53)	31 (43)	25 (76)	**0.002**	30 (45)	26 (68)	**0.020**
≥ 3 B lines	17 (16)	7 (10)	10 (30)	0.116	8 (12)	9 (24)	0.116
Any confluent B-line(s)	97 (92)	64 (89)	33 (100)	0.054	60 (90)	37 (97)	0.253
Small (<1 cm) confluence	46 (44)	35 (49)	11 (33)	0.143	35 (52)	11 (29)	**0.021**
Large (>1 cm) confluence	92 (88)	59 (82)	33 (100)	**0.008**	56 (84)	36 (95)	0.127
White lung	40 (38)	21 (29)	19 (58)	**0.005**	21 (31)	19 (50)	0.059
Non-homogenous pleural line	82 (78)	54 (75)	28 (85)	0.257	50 (75)	32 (84)	0.254
Any consolidation(s)	57 (54)	32 (44)	25 (76)	**0.003**	32 (48)	25 (66)	0.075
Small (< 1 cm)	45 (43)	24 (33)	21 (64)	**0.004**	22 (33)	23 (61)	**0.006**
Large (> 1 cm)	33 (31)	18 (25)	15 (45)	**0.036**	21 (31)	12 (32)	0.980
Total ultrasound score	12 (6, 18)	8.5 (4, 15)	18 (11, 22)	**<0.001**	10.0 (4, 18)	15.0 (8, 21)	**0.016**
	All patients n = 105 (%)	Alive n = 96 (%)	Death n = 9 (%)	p – value	No ICU (n = 80)	ICU (n = 25)	p - value
Abnormal lung ultrasound	97 (92)	88 (92)	9 (100)	1.000	72 (90)	25 (100)	0.194
Distribution							
Bilateral	80 (76)	71 (74)	9 (100)	0.111	55 (69)	25 (100)	**<0.001**
Affected areas ≥ 6	56 (53)	50 (52)	6 (67)	0.498	39 (49)	17 (68)	0.092
≥ 3 B lines							
Any confluent B-line(s)	97 (92)	88 (92)	9 (100)	1.000	72 (90)	25 (100)	0.194
Small (<1 cm) confluence	46 (44)	42 (44)	4 (44)	1.000	37 (46)	9 (36)	0.367
Large (>1 cm) confluence	92 (88)	83 (87)	9 (100)	0.597	67 (84)	25 (100)	**0.035**
White lung	40 (38)	35 (37)	5 (56)	0.297	25 (31)	15 (60)	**0.010**
Non-homogenous pleural line	82 (78)	75 (78)	7 (78)	1.000	62 (78)	20 (80)	0.792
Any consolidation(s)	57 (54)	51 (53)	6 (67)	0.504	40 (50)	17 (68)	0.115
Small (< 1 cm)	45 (43)	41 (43)	4 (44)	1.000	31 (39)	14 (56)	0.128
Large (> 1 cm)	33 (31)	30 (31)	3 (33)	1.000	23 (29)	10 (40)	0.290
Total ultrasound score	12 (6, 18)	12 (5, 18)	18 (10, 19)	0.122	11 (4, 17)	18 (10, 22)	**0.004**

Values are N (%) or Median (IQR). Intensive respiratory support defined as high flow oxygen via nasal cannula, a non-rebreather mask, or mechanical ventilation.

* ICU care, mechanical ventilation, or death are not mutually exclusive.

#### Lung findings predicting clinical outcomes

**Figure 3.**
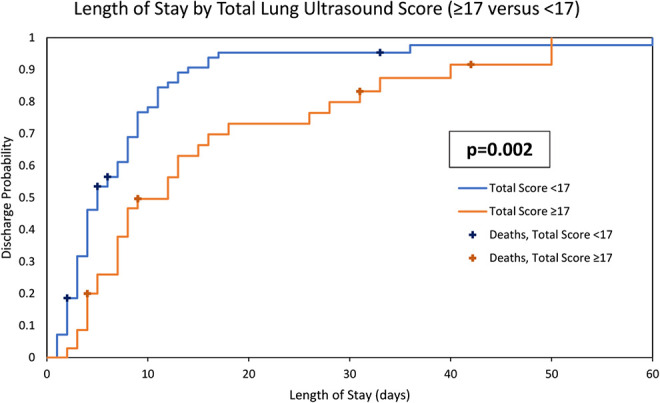
Cumulative incidence of duration of length of stay by total lung ultrasound score. Stratified by the lower 2/3 of total lung ultrasound scores (<17) versus the upper 1/3 (≥17).

The total lung ultrasound score ranged from 0 to 25 with each third of patients having a total lung ultrasound of 0-7, 8-16 and 17-25, respectively. The total lung ultrasound 0-7 score group had a median LOS of 4 days, the total lung ultrasound score 8-16 group median was 5 days and the highest total lung ultrasound score group 17-25 had a median hospital stay of 9 days. The total lung ultrasound scores for the shortest two-thirds length of stays (0-16 score) compared to upper third (≥17) was significantly different (p = 0.002) and is illustrated in **Figure 3**.

Logistic regression models built using all twelve lung zones could not predict any of the pre-specified outcomes; however, models with a subset of zones were more predictive. The best model using lung ultrasound scores included only 6 zones: L1, R1, L3, R3, L6, and R6. This model did not well predict death (AUC 0.69) or length of stay ≥9d (AUC 0.66). However, it better predicted need for intensive respiratory support (AUC 0.76) or ICU need (AUC 0.81) and a negative prediction had a NPV of 89% (95% CI 80-95%) for ICU need and 94% (95% CI 83-99%) for requiring intensive respiratory support Two zones scores stood out as most predictive, L6 = 3 and R1 ≥2 for respiratory support or ICU need, and predictive odds of each were significant (P < 0.01).

Similarly, using individual findings from a more limited 4 zones (L1, R1, L6, and R6) had moderate predictability for hospitalizations ≥ 9d (AUC 0.7), intensive respiratory support (AUC 0.77), or ICU (AUC 0.82) but not death (AUC 0.35). Similar to the lung ultrasound model, negative prediction had a NPV of 89% (95% CI 81-95%) for ICU need, 86% for hospitalizations ≥ 9d (95% CI 71- 95%) and 88% (95% CI 78-95%) for requiring intensive respiratory support. Again, the R1 and L6 zones findings had significant odds related to outcomes. Specifically, large consolidation in L6 had significant odds for predicting hospitalizations ≥9 days, respiratory support, or ICU need. Confluence is what seemed to matter in R1 with large confluence having significant odds of respiratory support or ICU need (P > 0.01). Full logistic regression operating characteristics are shown in in supplementary Tables S1.and S2, coefficients and odds are shown supplementary Tables S3.and S4 and an online calculator is available for real-time predictions (http://goodybedside.com/pocus/covidcalculator).

#### Other radiography

Chest radiographs were abnormal in 78% (80/103). Of 23 participants with normal chest radiographs, 17 had abnormalities detected on lung ultrasound. The abnormalities discovered by ultrasound included confluent B-lines (n = 16), pleural irregularity (n = 11), or small consolidations (n = 2). The negative predictive value of a normal chest radiograph was 26% (6/23) for not finding lung abnormalities on ultrasound. Due to infection control considerations, only 14 participants had received CT of the chest.

## Discussion

We conducted a prospective observational cohort study at 2 tertiary care hospitals in the United States on consecutive patients with confirmed COVID-19 infection admitted to a non-ICU hospital medicine service. We endeavored to describe in detail the lung ultrasound findings in this cohort as well as to determine if any of those findings are associated with or predictive of predefined clinical outcomes.

Most of the patients (92%) had abnormal findings detected on point-of-care ultrasound. Confluent B-lines, non-homogenous pleural lines, consolidations, and bilateral involvement were frequent findings in hospitalized patients with COVID-19, consistent with prior reports.^10,15,16,25^ While these findings are not specific to COVID-19 pneumonia, these findings can aid in the diagnosis of COVID-19 pneumonia when the clinical suspicion is high.

Large confluent B-lines, consolidations, bilateral involvement, and any abnormality in ≥ 6 areas were associated with a longer hospitalization and need for intensive respiratory support. Large confluent B-lines and bilateral involvement were also associated with ICU stay. Any confluent B-line in R1 had increased odds for IRS and ICU need in our logistic regression model. That anterior and upper zone findings were prognostic is in line with the findings of Castelao^16^ and involvement of upper lung zones on CT has been associated with worse clinical outcome.^26^ But that our finding is limited to the right anterior zone is difficult to explain physiologically and may be a stochastic anomaly, despite our larger patient cohort.

That consolidation is a late COVID-19 finding has been previously described^7^ but surprisingly large consolidations were only statistically associated with the intensive respiratory support group, not length of stay, or death. We observed large consolidations with nearly equal frequency at either base (L6 and R6); however, only a large consolidation in L6 was prognostic, not one in R6. Large consolidations in L6 zone had significant odds for a longer hospitalization or need for intensive respiratory support and ICU. Again, the unilateral nature of this finding is difficult to explain, but that these outcome groups were not small (ICU n = 25/105, respiratory support 33/105, hospitalization ≥9d 38/105) and the result was significant and tested on an independent subset of our data is compelling. Given that large consolidation in R6 had little influence on the outcome prediction (instead of relatively lesser influence) increases the suspicion that there may be something to this unilateral observation. A similar observation was reported by Yu et al. looking at the prognostic value of CT findings on admission. They described an increased incidence of left lower lobe consolidation with worse outcome, though the result was not statistically significant.^26^

The lung ultrasound score has good reproducibility (κ = 0.95). Previous studies on lung ultrasound reported that higher scores were associated with need for high-flow nasal cannula, continuous positive airway pressure, noninvasive mechanical ventilation,^16^ hospitalization,^15^ intensive care stay,^14^ mechanical ventilation and death.^17^ In our study, higher total lung ultrasound scores were associated with need for intensive respiratory support or ICU, and a total lung ultrasound score of ≥17 associated with a longer length of stay (9 vs. 5 d, p = 0.002). The ultrasound score in lung zones R1 and L6 was prognostic, with R1 ≥ 2 and L6 = 3 having significant odds for the need for intensive respiratory support or ICU but not predictive of longer hospitalization or death. No finding seemed to be associated with death though 9% mortality rate in a cohort of 105 patients may be too low to observe a difference. Also, the deaths were sporadic, and cause of death from COVID-19 is often not purely from respiratory complications, while lung ultrasound findings likely primarily relate to respiratory status.

Our study has notable limitations. First, we excluded patients who were unable to provide consent. Neurologic manifestations such as impaired consciousness are common in patients with severe COVID-19.^27^ Therefore, some patients with more severe disease may have been excluded. Second, we used a phased-array probe, which is commonly used in hospital medicine to perform ultrasound examinations. Lung ultrasound using a higher frequency probe (e.g., linear probe) with shallower depth is likely more sensitive in detecting more subtle pleural line abnormalities and smaller consolidations, though it is possible it is too sensitive as Haaksma et al. previously demonstrated that the linear probe had worse agreement compared to a phased array probe.^28^ Additionally, examination using 2 different probes for lung ultrasound in COVID-19 patients is not practical and may increase the examination time and the risk of exposure. Finally, we did not perform serial ultrasounds. It is unclear if the change in total lung ultrasound scores over time may have better prognostic value.

In conclusion, most of the patients hospitalized with COVID-19 had lung ultrasound abnormalities on admission and a higher ultrasound score was associated with worse clinical outcomes, longer LOS, but not with death. Point-of-care ultrasound can aid in the risk stratification for patients with COVID-19 admitted to internal medicine wards in order to triage those at higher risk of needing further supportive care or interventional therapy. Additionally, our models appear to predict which patients will not have poor outcomes, excluding death, but this needs to be prospectively validated.
